# Effect of Anti-Hp treatment on nutritional status of children with Helicobacter Pylori-Positive Gastritis and its clinical significance

**DOI:** 10.12669/pjms.37.5.4234

**Published:** 2021

**Authors:** Na-ying Zuo, Yuan-da Zhang, Qing-wei Dong, Jing Bi, Xiao Liu

**Affiliations:** 1 Na-ying Zuo, Department of Gastroenterology, Baoding Children’s Hospital, Baoding, Hebei, 071000, P.R. China. Key Laborary of Clinical Research on Respiratory Digestive Disease, Hebei Baoding, 071000, China; 2 Yuan-da Zhang, Department of Gastroenterology, Baoding Children’s Hospital, Baoding, Hebei, 071000, P.R. China. Key Laborary of Clinical Research on Respiratory Digestive Disease, Hebei Baoding, 071000, China; 3 Qing-wei Dong, Department of Gastroenterology, Baoding Children’s Hospital, Baoding, Hebei, 071000, P.R. China. Key Laborary of Clinical Research on Respiratory Digestive Disease, Hebei Baoding, 071000, China; 4 Jing Bi, Department of Infectious Diseases, Baoding Children’s Hospital, Baoding Children’s Hospital, Baoding, Hebei, 071000, P.R. China; 5 Xiao Liu, Department of Gastroenterology, Baoding Children’s Hospital, Baoding, Hebei, 071000, P.R. China. Key Laborary of Clinical Research on Respiratory Digestive Disease, Hebei Baoding, 071000, China

**Keywords:** Helicobacter pylori, gastritis, malnutrition, children, treatment

## Abstract

**Objectives:**

To evaluate the outcome of anti-HP treatment on the nutritional status of children with Helicobacter pylori-positive gastritis.

**Methods:**

Sixty children with Helicobacter pylori-positive gastritis admitted to our hospital from June 2018 to June 2020 were selected as the experimental group, and 60 healthy normal people (Hp negative) were selected as the control group. The experimental group were given anti-HP treatment, and the improvement of their clinical symptoms after treatment and the changes of nutritional indexes such as hemoglobin and serum ferritin were observed one year after treatment. Gastroscopy was performed before treatment and four weeks after treatment, and the improvement of gastric inflammation and the positive rate of Hp were compared and analyzed before and after treatment.

**Results:**

The nutritional indicators of the children in experimental group were inferior compared with those in the control group (p<0.05). The clinical symptoms and signs of the experimental group were significantly alleviated after anti-Hp treatment, and the biochemical indicators were significantly improved after one Year of follow-up compared with those before treatment (p<0.05). The incidence of moderate and severe gastric mucosal inflammation in the experimental group decreased from 70% before treatment to 17% (p<0.05). The HP infection decreased from 100% before treatment to 13% (p<0.05).

**Conclusion:**

Helicobacter pylori infection has a negative impact on the nutritional status of children. Anti-HP therapy can improve the gastrointestinal symptoms and nutritional status of children, which plays an important role in the growth and development of children.

## INTRODUCTION

Gastritis, as a common clinical disease of the digestive system in children, can lead to a series of complications such as water electrolyte disorder and malnutrition. With the continuous progress of examination technology and means, Helicobacter pylori (HP) infection has been found to be the main cause of gastritis in children.^1^ HP is one of the more common pathogens in the human digestive system. About half of the world’s population is infected with HP, but only a portion of Hp infected individuals develop gastric and duodenal lesions. It is therefore believed that the pathogenesis of Hp infection and disease outcomes is mediated by a complex interaction between host, environment and bacterial virulence factors.^2^

HP infection mainly develops in childhood.^3^ Especially in children in developing countries, the incidence can be as high as 30% or more^4^, and gradually increases with age. Children infected with Hp will suffer from gastric mucosal congestion, edema, and erosion. As a result, nutrients and trace elements cannot be absorbed normally, which eventually leads to malnutrition in children and affects normal development.^5^ According to the study of Dror et al.^6^ Hp infection will have an adverse effect on the growth of children, and the eradication of Hp infection can lead to bone growth and weight gain of children, as well as improve the nutritional status. In this study, 60 children with Hp positive gastritis were treated with anti-Hp treatment, and their clinical symptoms, Hp negative status, and changes in nutritional indicators were followed up after treatment. Our objective was to verify the significance of anti-Helicobacter pylori therapy in improving the nutritional status and clinical efficacy of children with Helicobacter pylori positive gastritis.

## METHODS

Sixty cases of children with Helicobacter pylori-positive gastritis admitted to our hospital from June 2018 to June 2020 were selected as the experimental group, and 60 cases of healthy normal people (Hp negative) were selected as the control group. The study was approved by the Institutional Ethics Committee of Baoding Children’s Hospital on June 10, 2020(No. H-BDETKJ-SOP006-03-A/0), and written informed consent was obtained from all participants. Thirty five males and 25 females were grouped into the experimental group, aged 4 - 14 years old, with an average of 7.3 ± 2.4 years old. 37 males and 23 females were grouped into the control group, aged 4 - 14 years old, with an average of 7.6 ± 2.5 years old. The baseline data of the two groups were balanced and comparable (p>0.05).

### Inclusion criteria:

1. Children with digestive diseases but without severe organic or congenital diseases of heart, liver and kidney.
2. Children aged between 4 - 14 yesars old.
3. Children who have tested positive for Helicobacter pylori[7]: C13-UBT test were both.
4. Children with clinical manifestations and physical examination showing tenderness in the upper abdominal wall or around the umbilicus.
5. Children diagnosed with chronic gastritis by gastroscopy.^8^
6. Children who can cooperate with and accurately describe subjective symptoms.
7. Children whose families are willing and able to cooperate to complete the study and sign the informed consent form.

### Exclusion criteria:

1. Children with nutritional deficiency due to other reasons.
2. Children with mesenteric inflammation and other diseases of liver, gallbladder, pancreas and spleen.
3. Children who dropped out of the study.

General treatment includes actively symptomatic treatment, correction of unhealthy living or eating habits, and avoidance of cold and spicy food.

### Hp eradication regimen^9^

Children aged 4-6 were treated with standard triple therapy: Omeprazole 0.6-1.0mg/(kg·d), twice before meals for 2 weeks; amoxicillin 50mg/(kg·d), with the maximum dose of one g for two weeks; clarithromycin 15-20 mg/(kg·d), with the maximum dose of 0.5 g for two weeks. Children over six years old were treated with a modified quadruple anti-Hp regimen, that is, bismuth agent was added on the basis of standard triple therapy: 6-8 mg/(kg·d), orally taken twice before meals for 7d. All the children underwent gastroscopy before and 4 weeks after treatment, followed up for one year after treatment, and their changes in various nutritional status indicators before and after treatment were recorded.

The differences in nutritional indicators such as height, weight, hemoglobin and serum iron between the experimental group and the healthy control group were compared and analyzed. Improvement of clinical symptoms: the changes of symptoms and signs such as abdominal pain, nausea, vomiting and abdominal tenderness were recorded before and 4 weeks after treatment in the experimental group. The clinical manifestations of abdominal pain, nausea, vomiting and abdominal tenderness were scored with Gastrointestinal Symptom Rating Scale (GSRS)^10^ according to the severity of symptoms, with 0, two, four and six months. The higher the score, the more serious the symptoms.The changes in nutritional indicators such as hemoglobin and serum ferritin before and one year after treatment were compared and analyzed.

### Pathological analysis of gastroscopy

Gastroscopy was performed before and four weeks after treatment. 1-2 specimens were collected from the sites with inflammation, and one specimen was taken from the large and small curvature of the gastric antrum. The specimens were fixed in 2% formaldehyde, and the degree of inflammation was evaluated by HE staining, which was divided into none, mild, moderate and severe.^11^ Hp kit was utilized for the rapid urease test, and a positive result indicated Hp infection. The improvement of gastric inflammation and Hp positive rate before and after treatment were compared and analyzed.

### Statistical Analysis

All the data were statistically analyzed by SPSS 20.0 software, and the measurement data were expressed as (X̅±s). Two tailed independent student’s t-test was used for inter-group data analysis, paired t-test was used for intra-group data analysis, and c^2^ was adopted for rate comparison. P<0.05 indicates a statistically significant difference.

## RESULTS

The comparative analysis of the nutritional indicators between the experimental group and the control group is shown in Table-I, indicating that the nutritional indicators of children in the experimental group were inferior to varying degrees compared with those in the healthy control group, and that the children with Hp-positive gastritis had impaired nutritional status.

**Table-I:** Comparative analysis of nutritional indicators between the experimental group and the control group ( X̅±S) n=60.

Indicators	Average age(y)	Height (cm)*	Weight (kg)*	Hemoglobin (g/L)*	Serum iron (mmol/L)*
Experimental group	7.3±2.4	122.0±14.9	24.1±9.3	121.0±7.4	12.2±2.2
Control group	7.6±2.5	128.2±16.1	27.8±10.6	135.1±7.2	13.9±2.2
t	0.954	2.198	2.034	10.518	4.169
P	0.327	0.030	0.044	0.00	0.00

* P<0.05

In the experimental group, the clinical symptoms and signs were significantly relieved after anti-HP treatment, which was specifically characterized by a significant improvement in abdominal pain, nausea, vomiting, and abdominal tenderness, etc., with a statistically significant difference (p<0.05) (Table-II).

**Table-II:** Comparative analysis of symptoms before and after treatment in the experimental group (X̅ ±S) n=60.

Group	Abdominal pain*	Nausea*	Vomiting*	Abdominal tenderness*
Before treatment	2.7±1.0	1.0±1.2	0.5±1.0	3.0±1.0
After treatment	0.7±1.0	0.3±0.8	0.2±0.6	0.9±1.0
t	11.014	3.367	2.157	11.237
p	0.00	0.001	0.034	0.00

* p<0.05.

Children in the experimental group were re-examined one year after anti-Hp treatment, and their various indicators such as hemoglobin, serum iron and ferritin were significantly improved compared with those before treatment, with a significant difference (p<0.05) (Table-III).

**Table-III:** Comparative analysis of serum biochemical indicators before and after treatment in the experimental group (X̅ ±S) n=60.

Group	Hemoglobin (g/L)*	Serum iron (mmol/L*	Ferritin (ug/L)*
Before treatment	121.0±7.4	12.2±2.2	27.6±6.5
After treatment	133.3±9.1	13.7±2.9	33.1±4.2
t	8.162	3.090	5.616
p	0.00	0.003	0.00

* p<0.05

The pathological results of gastroscopy in the experimental group after treatment showed that: the incidence of moderate to severe gastric mucosal inflammation before anti-HP treatment was 70% and decreased to 17% after treatment. Gastric mucosal inflammation improved significantly after treatment, which was statistically significant (p<0.05); The rapid urease test showed that Hp infection decreased from 100% before treatment to 13% after treatment, and the improvement was significant before and after treatment (p<0.05), with a statistical significance (Table-IV).

**Table-IV:** Comparative analysis of pathological results of gastroscopy before and after treatment in the experimental group (X̅ ±S) n=60.

Indicators	Incidence of gastric mucosal inflammation					Hp-positive rate

	None	Mild	Moderate	Severe	Moderate to severe incidence	
Before treatment	0	18	27	15	42(70%)	60(100%)
After treatment	45	5	7	3	10(17%)	8(13%)
c^2^					34.751	91.765
P					0.00	0.00

p<0.05.

## DISCUSSION

Helicobacter pylori (Hp) infection is declining worldwide in terms of its incidence, especially among children in developed countries, but still has a high level in children in some developing countries.^12^ Children with HP will suffer from chronic gastritis and peptic ulcers, and in severe cases, malignant tumors of the digestive tract.^13^ Hp infection is mainly transmitted via the oral cavity. It was concluded in the study of Batawi et al.^14^ that HP exists in the oral cavity of children with dental caries. Dental caries may become the reservoir of HP, so as to gradually cause gastric mucosal inflammation, congestion, edema and other symptoms as food enters the stomach and settles in the stomach. As a result, energy intake will be affected and symptoms such as gastric nutrient absorption disorders will progress, which directly affects the growth and development of children.^15^

HP infection will affect the serum ferritin concentration in children, resulting in iron deficiency and iron deficiency anemia, thereby affecting the growth of children.^16^ The results of this study also confirmed that the serum iron concentration of Hp-positive children was significantly lower than that of normal children. Furthermore, HP infection will cause anorexia^17^ and reduced dietary intake in children, further increasing the occurrence and development of malnutrition. In recent years, such a pathogen has been shown in numerous studies to cause malabsorption of several nutrients such as cobalamin, vitamin C and vitamin E, and has a strong impact on nutritional status.^18^ Studies have shown that H. pylori infection impair the absorption of iron and vitamin B12, promotes the secretion of anorexia hormones, and may eventually lead to growth retardation in younger children.^19^ It is suggested in this study that nutritional indicators such as height, weight, hemoglobin and serum iron in the experimental group were significantly lower than those in the healthy control group, suggesting that children with HP-positive gastritis have abnormal nutritional status. The results are similar to those of previous studies.

It is considered by Venneman et al.^20^ that eradication of Hp may be beneficial to the improvement of nutritional status. And elimination of HP infection is the most important approach for the treatment of gastritis in children.^21^ Standard anti-HP therapy needs to be taken^22^ for treatment given the very small chance of spontaneous regression of Hp infection.^23^ Currently, triple therapy (omeprazole, amoxicillin, clarithromycin) or bismuth quadruple therapy (omeprazole, bismuth, amoxicillin, clarithromycin) are the main methods to treat the disease. The existing therapies are adjusted on the basis of omeprazole, and the resulting therapeutic effects are similar.^24^ This study refers to the treatment ideas of scholars, children over 6 years old are treated with modified quadruple therapy: omeprazole, bismuth, amoxicillin, clarithromycin. And results shows that the degree of gastric mucositis is significantly reduced after anti-HP therapy, the clinical symptoms and signs of the children were better than those before treatment. It has been proved that quadruple therapy is effective in the radical treatment of HP infection in children.

### Limitations of this study

Nevertheless, the shortcomings can still be seen in this study: only the older children were selected because they could accurately describe the main symptoms so as to make the study results more accurate and objective. Consequently, the therapeutic effect for children of younger age has not been evaluated. In addition, children are followed up for as short as one year, and the long-term therapeutic effect of anti-Hp treatment on children with Hp-positive gastritis and the long-term nutritional recovery status has not yet been reflected. In view of this, further countermeasures are being taken to increase sample size and conduct long-term follow-up, especially to increase the number of younger children and related studies, with a view to a more detailed and objective evaluation of the clinical significance of anti-Hp treatment in children with Hp-positive gastritis.

## CONCLUSION

Helicobacter pylori infection has a negative impact on the nutritional status of children. Anti-HP therapy can improve the gastrointestinal symptoms and nutritional status of children, which plays an important role in the growth and development of children.

### Authors’ Contributions:

**NYZ & YDZ:** designed this study and prepared this manuscript, and responsible for the accuracy and integrity of the work.

**QWD& JB:** Collected and analyzed clinical data.

**XL:** Significantly revised this manuscript.

## References

[1] Ushiku T, Moran CJ, Lauwers GY (2013). Focally enhanced gastritis in newly diagnosed pediatric inflammatory bowel disease. Am J Surg Pathol.

[2] Sterbenc A, Jarc E, Poljak M, Homan M (2019). Helicobacter pylori virulence genes. World J Gastroenterol.

[3] George S, Lucero Y, Torres JP, Lagomarcino AJ, O'Ryan M (2020). Gastric Damage and Cancer-Associated Biomarkers in Helicobacter pylori-Infected Children. Front Microbiol.

[4] Jin Y, Zhang S, Pan J, Yue M, Zhang G, Yao D (2019). Comparison of efficacy and safety of ilaprazole and esomeprazole both in initial treatment regimen and retreatment regimen of Helicobacter pylori infection in chronic gastritis. Pharmazie.

[5] Sugimoto M, Yasuda H, Andoh A (2018). Nutrition status and Helicobacter pylori infection in patients receiving hemodialysis. World J Gastroenterol.

[6] Dror G, Muhsen K (2016). Helicobacter pylori Infection and Children's Growth:An Overview. J Pediatr Gastroenterol Nutr.

[7] Sabbagh P, Javanian M, Koppolu V, Vasigala VR, Ebrahimpour S (2019). Helicobacter pylori infection in children:an overview of diagnostic methods. Eur J Clin Microbiol Infect Dis.

[8] Bacha D, Walha M, Ben Slama S, Ben Romdhane H, Bouraoui S, Bellil K (2018). Chronic gastritis classifications. Tunis Med.

[9] Yang JC, Lu CW, Lin CJ (2014). Treatment of Helicobacter pylori infection:current status and future concepts. World J Gastroenterol.

[10] Ghalichi F, Ghaemmaghami J, Malek A, Ostadrahimi A (2016). Effect of gluten free diet on gastrointestinal and behavioral indices for children with autism spectrum disorders:a randomized clinical trial. World J Pediatr.

[11] Lahner E, Zagari RM, Zullo A, Di Sabatino A, Meggio A, Cesaro P (2019). Chronic atrophic gastritis:Natural history, diagnosis and therapeutic management. A position paper by the Italian Society of Hospital Gastroenterologists and Digestive Endoscopists [AIGO], the Italian Society of Digestive Endoscopy [SIED], the Italian Society of Gastroenterology [SIGE], and the Italian Society of Internal Medicine [SIMI]. Dig Liver Dis.

[12] Burucoa C, Axon A (2017). Epidemiology of Helicobacter pylori infection. Helicobacter.

[13] Luan C, Liu Z, Li Y, Dong T (2020). Association among helicobacter pylori infection, gastrin level and colorectal cancer in patients aged 50 years and over. Pak J Med Sci.

[14] El Batawi HY, Venkatachalam T, Francis A, Abujabal R, Shehadat SA (2020). Dental Caries-A Hiding Niche for Helicobacter Pylori in Children. J Clin Pediatr Dent.

[15] Kalach N, Bontems P, Raymond J (2017). Helicobacter pylori infection in children. Helicobacter.

[16] Bibi F, Alvi SA, Sawan SA, Yasir M, Sawan A, Jiman-Fatani AA (2017). Detection and Genotyping of Helicobacter pylori among Gastric ulcer and Cancer Patients from Saudi Arabia. Pak J Med Sci.

[17] Savoldi A, Carrara E, Graham DY, Conti M, Tacconelli E (2018). Prevalence of Antibiotic Resistance in Helicobacter pylori:A Systematic Review and Meta-analysis in World Health Organization Regions. Gastroenterology.

[18] Aimasso U, D'onofrio V, D'eusebio C, Devecchi A, Pira C, Merlo FD (2019). Helicobacter pylori and nutrition:a bidirectional communication. Minerva Gastroenterol Dietol.

[19] Franceschi F, Annalisa T, Teresa DR, Giovanna D, Ianiro G, Franco S (2014). Role of Helicobacter pylori infection on nutrition and metabolism. World J Gastroenterol.

[20] Venneman K, Huybrechts I, Gunter MJ, Vandendaele L, Herrero R, Van Herck K (2018). The epidemiology of Helicobacter pylori infection in Europe and the impact of lifestyle on its natural evolution toward stomach cancer after infection:A systematic review. Helicobacter.

[21] Miguel N, Costa E, Santalha M, Lima R, Vizcaino JR, Pereira F (2014). Refractory iron-deficiency anemia and autoimmune atrophic gastritis in pediatric age group:analysis of 8 clinical cases. J Pediatr Hematol Oncol.

[22] Goderska K, Agudo Pena S, Alarcon T (2018). Helicobacter pylori treatment:antibiotics or probiotics. Appl Microbiol Biotechnol.

[23] Goderska K, Agudo Pena S, Alarcon T (2018). Helicobacter pylori treatment:antibiotics or probiotics. Appl Microbiol Biotechnol.

[24] de Brito BB, da Silva FAF, Soares AS, Pereira VA, Santos MLC, Sampaio MM (2019). Pathogenesis and clinical management of Helicobacter pylori gastric infection. World J Gastroenterol.

